# Understanding tobacco use and socioeconomic inequalities among men in Ghana, and Lesotho

**DOI:** 10.1186/s13690-017-0197-5

**Published:** 2017-07-03

**Authors:** Kwamena Sekyi Dickson, Bright Opoku Ahinkorah

**Affiliations:** 1 Department of Population and Health, University of Cape Coast, Cape Coast, Ghana; 2 Department of Health, Physical Education and Recreation, University of Cape Coast, Cape Coast, Ghana

**Keywords:** Tobacco use, Socio – economic, Inequalities, Ghana, Lesotho

## Abstract

**Background:**

Tobacco use is one of the leading causes of preventable deaths and has become a significant public health issue. Previous studies have paid less attention to tobacco use and socio-economic equalities among men in developing countries. This study examines the relationship between tobacco use and socio-economic inequalities among men in Ghana and Lesotho.

**Methods:**

The study made use of data from the 2014 Demographic and Health Survey (DHS) from Ghana, and Lesotho. Binary logistic regression was employed to examine the associations between socio-economic inequality characteristics of respondents and tobacco use.

**Results:**

The results showed that the prevalence of tobacco use was high in Lesotho (47.9%) as compared to that of Ghana (6.3%). Tobacco use was generally high across all age groups in Lesotho and in contrast, it was relatively low across all ages in Ghana. A statistically significant association was found between all the socio-economic variables and tobacco use in both countries. The prevalence of tobacco use was smaller in age group 15–24 years compared to the age groups 25–34 years and 35–59 years in both Ghana and Lesotho, although the association is stronger in Ghana. The AOR’s in Ghana are respectively 5.3 (95% CI: 3.29–8.59) and 9.7 (95% CI: 6.20–15.06), compared to respectively 1.7 (95% CI: 1.32–2.11) and 1.7 (95% CI: 1.36–2.12). Smoking prevalence was smaller in men with higher level of education compared to men with no education in both Ghana and Lesotho, although the association was weaker in Ghana. The AOR in Ghana is 0.1 (95% CI: (0.02–0.11), compared to 0.2 (95% Cl: (0.17–0.30). The prevalence of tobacco use was smaller among men in urban areas compared to rural areas in both Ghana and Lesotho, although the association is stronger in Ghana. The AOR in Ghana is 2.1 (95% CI: 1.67–2.73), compared to 1.6 (95% CI: (1.31–1.95). In both countries, prevalence of tobacco use was higher in men who are traditionalist/spiritualists or who had no religion compared to Christians, although the association was stronger in Ghana. The AOR in Ghana is 6.2 (95% CI: (4.42–4.09) compared to 1.7 (95% CI: (1.21–2.47). The prevalence of tobacco use was low among men with richest wealth status compared to men with poorest wealth status in both Ghana and Lesotho, although the association is weaker in Ghana. The AOR in Ghana is 0.1 (95% Cl: (0.06–0.17) compared to 0.4 (95% CI: (0.51–1.12). In relation to occupation, prevalence of tobacco use was smaller among professional workers compared to men in the Agricultural sector in both Ghana and Lesotho, although the association is stronger in Ghana. The AOR in Ghana is 9.3 (95% Cl: (4.54–18.99), compared to 3.5 (95% CI: (2.27–5.52). Formerly married men in both countries were more likely to use tobacco compared to currently not married men, although the prevalence was higher in Ghana. The AOR in Ghana is 1.6 (95% CI: (0.99–2.28)], compared to 1.4 (95% CI: (0.89–2.28) in Lesotho.

**Conclusion:**

Although similar socio-economic inequality factors provided an understanding of tobacco use among men in Ghana and Lesotho, there were variations in relation to how each factor influences tobacco use.

**Electronic supplementary material:**

The online version of this article (doi:10.1186/s13690-017-0197-5) contains supplementary material, which is available to authorized users.

## Background

Tobacco consumption has been identified as a global public health issue and a major cause of premature mortality and morbidity [1]. It has been seen as a cause of suffering and socio-economic problems in humans [2]. Globally, tobacco use is one of the leading causes of preventable deaths and has become a significant public health concern [3–5]. Direct tobacco use is estimated to cause five million deaths a year globally, while indirect exposure leads to an additional 600, 000 deaths [6]. With the implementation of smoke-free policies in most developed countries, the general prevalence of smoking has declined, but rates remain particularly high among lower socioeconomic groups, including those with lower education levels, incomes, and employment status [7–9]. Among men, the relatively high prevalence of tobacco use in the lowest socioeconomic groups can in part explain the socioeconomic inequalities in health in most developed countries where socioeconomic inequalities in tobacco use contribute to socioeconomic inequalities in mortality [10, 11].

Health behaviours, and the inequitable distribution of such determinants of population health, influence the future incidence of certain common chronic diseases and thus have a considerable impact on health status and utilization of health care services and costs. Estimates from World Health Organization (WHO) indicate that 37% of the burden of disease in Western Europe is attributable to tobacco smoking, alcohol consumption, diet and high cholesterol, physical inactivity and overweight [12]. Particularly tobacco smoking contributes to a large amount of the burden of disease in high income countries. Since this factor in most cases is distributed in a socioeconomically inequitable manner, it also contributes significantly to health inequalities in the mentioned part of the world [13]. Tobacco use is the leading preventable cause of death and disability worldwide [14]. Smoking and tobacco use cause 5.4 million deaths annually [15] and have been associated with numerous cancers including lung cancer, pancreatic cancer, cancer of the larynx, and cervical cancer [16]. Smoking and tobacco use are also associated with chronic diseases and other adverse health outcomes, including stroke, coronary heart disease, chronic obstructive pulmonary disease (COPD), periodontitis, hip fractures, pneumonia, and reduced fertility among women [16].

The prevalence of tobacco use varies substantially across regions and across countries. Some of the highest prevalence of cigarette smoking among men is found in East Asia, Southeast Asia, Eastern Europe, and Central Asia [17]. According to data published in the WHO Global Status Report on Non communicable Diseases [14], countries with more than a 50% prevalence of tobacco smoking among men include Armenia, Belarus, the Democratic People’s Republic of Korea, Greece, Indonesia, Kiribati, Papua New Guinea, the Republic of Korea, the Russian Federation, Samoa, Tunisia, and Ukraine. As a result of the stronger tobacco control environments and falling tobacco use in most high-income countries and the increased globalization that has increasingly opened markets in low-and middle-income countries, multinational tobacco companies have significantly expanded their presence in developing countries, which are still in the early stages of the tobacco epidemic [18, 19]. With the majority of new tobacco users coming from low- and middle-income countries, and tobacco use growing faster in those countries compared to high-income countries, the burden of death and disease caused by tobacco use is shifting to the developing world [20]. Using a population-based data from 16 Demographic Health Surveys (DHS) of men aged 15–54 years and women aged 15–49 years in 14 nations in Africa, Pampel [21] found that the ranking of the nations on the use of pipes and other forms of smoking tobacco differs across countries. Zambia (10.4%), Namibia (10.6%), Mozambique (11.9%), Madagascar (17.7%), and Lesotho (25.1%) have the highest usage, while Ghana, Nigeria, Ethiopia, Kenya, Zimbabwe, and Tanzania have usage under 2%.

The negative health effects of smoking are not limited to smokers [22]. Globally, it has been estimated that exposure to secondhand smoke is responsible for more than 600,000 deaths per year, including 166,000 deaths among children [23]. Oberg, Jaakkola et al. [23] also state that most deaths from secondhand smoke in adults are caused by ischemic heart disease, asthma, and lung cancer and in children most of these deaths are caused by lower respiratory infections. Secondhand smoke is also associated with sudden infant death syndrome [24] and lower birthweight [25]. Most research examining the adverse health effects of tobacco use has focused on manufactured cigarettes given their higher prevalence than other forms of tobacco. However, studies have also documented serious health consequences associated with the use of other types of smoked tobacco [26–29]. Moreover, there are few studies that have examined tobacco use and socio-economic inequalities among men. One study that specifically looked at tobacco use and socio-economic inequalities was conducted by Lakew and Haile [30]. Findings from the study showed that administrative region, wealth index, age, occupation, child death experience, religion, sex and marital status were significantly associated with tobacco use.

In most African countries, compared to other countries, much attention has not been paid to tobacco use and socio-economic equalities among men. Even though Doku, Darteh and Kumi-kyereme [31] conducted a study using the 2003 and 2008 Ghana Demographic and health survey report on socio-economic inequalities and cigarette smoking among men, the authors focused mainly on cigarette smoking, neglecting other forms of tobacco use. Again, it appears little has been done on the relationship between tobacco use and socio-economic inequalities among men across different countries in Africa. Therefore, this study is intended to examine the relationship between tobacco use and socio-economic inequalities among men in Ghana and Lesotho.

## Methods

### Data sources

Data from the 2014 Demographic and Health Survey (DHS) from Ghana and Lesotho were used for this paper. Demographic and Health Survey is a nationwide survey, which is designed and conducted every five years. The DHS focuses on child and maternal health and is designed to provide adequate data to monitor the population and health situation. DHS gathers information on maternal health, fertility, non-communicable disease and other health issues such as alcohol consumption, physical activity, health insurance coverage and the use of tobacco and much more. The Demographic and Health Surveys were carried out by the Ghana Statistical Service (GSS), in Ghana and the Ministry of Health (MOH) in Lesotho with technical support from ICF Macro through MEASURE DHS. In the 2014 version, 4388 men between the ages 15 and 59 from 12, 831 households covering 427 clusters were interviewed throughout Ghana and 2028 men aged 15–59 from 9402 households coving 400 clusters were interviewed in Lesotho. The response rate was 95.2% for Ghana, and 93.6% for Lesotho [32, 33]. For the purpose of this study, the sample used was 4372 for Ghana and 1836 for Lesotho. Permission to use the data set was given us by the MEASURE DHS following the assessment of a concept note. The dataset is available to the public (www.measuredhs.com).

### Study variables

The outcome variable employed for this study was tobacco use. The outcome variable was derived from the questions “do you currently smoke cigarette?” and “what (other) type of tobacco do you currently smoke or use?”. Five types of tobacco use were identified: chewing tobacco (yes, no), uses snuff (yes, no) smoke pipe (yes, no), smoke other (yes, no), smoke cigarette (yes, no). The ‘Yes’ responses were coded ‘1’ and the ‘No’ responses were coded ‘0’. An index was created with all the yes and no answers with scores ranging from 0 to 5. The score 0 was labelled as “non-users” and 1 to 5 was labelled as “users”. A dummy variable was generated with ‘0’ score being males who had not used any type of tobacco and ‘1’ if the males had used at least one type of tobacco.

Seven explanatory variables were used in the study – age, wealth status, education, residence, religion, occupation, and marital status. Age was recoded as 15–24, 25–34 and 35–59. Wealth status was categorized in poorest, poorer, middle, richer and richest. Education was classified into four categories: no education, primary education, secondary education and higher education. Religion was captured ad Christian, Muslim, traditional/spiritual/no religion and other. Occupation was captured as not working, professional, clerical, agriculture, services, skilled and unskilled. Marital status was recoded as currently not married (never married, and not living together, separated), married and formerly married (widowed, divorced). Type of residence was coded as urban or rural.

### Data analysis

Data analysis was carried out using STATA version 13. Since the outcome variable was a dichotomous variable, a discrete choice model was employed to show how the explanatory variables correlated with the outcome variable. Specifically, the binary logistic regression was employed given that this technique is more appropriate for dichotomous variables. A key assumption underlying the binary logistic regression model is that the dependent variable should be dichotomous in nature and the data should not have any outlier. The complex design used to collect the data were also built into the analysis to account for the two-stage design. A bivariate analysis was conducted for the outcome variable and explanatory variables and controlling for age. Next, a multivariable analysis was also conducted. Both the bivariate and multivariate analysis were conducted for the individual countries. Finally, both countries were appended together and an interaction between the two countries were looked at. These interactions were presented to show chi square, degree of freedom and *p* value.

## Results

The survey included weighted total male population of 4372, and 1836 in the age range 15–59 from Ghana and Lesotho respectively. The results showed that the prevalence of tobacco use was high in Lesotho (47.9%) as compared to that of Ghana (6.3%). Tobacco use was generally high across all age groups in Lesotho and in contrast, it was relatively low across age groups in Ghana. For instance, 1.6% of men in Ghana compared to 40.9% of men in Lesotho (see Table 1).

**Table 1:** Background characteristics and the prevalence of tobacco use among males 15 years and older in Ghana (GDHS 2014) and Lesotho (LDHS 2014)

Variables	Ghana *N* = 4372 (%)	Lesotho *N* = 1836 (%)
Tobacco use		
No	4096 (93.7)	956 (52.1)
Yes	276 (6.3)	880 (47.9)
Age		
15–24	23 (1.6)	252 (40.9)
25–34	70 (6.2)	292 (52.2)
35–59	183 (10.2)	336 (50.8)
Level of education		
No education	103 (21.7)	115 (65.3)
Primary	59 (9.9)	469 (54.7)
Secondary	106 (3.8)	257 (39.8)
Higher	8 (1.7)	39 (24.5)
Residence		
Urban	95 (4.2)	298 (42.4)
Rural	181 (6.3)	582 (51.3)
Religion		
Christian	112 (3.5)	792 (47.1)
Muslim	74 (9.6)	3 (35.2)
Traditional/spiritual/no religion	45 (27.8)	72 (58.9)
Other	45 (16.6)	13 (54.3)
Wealth status		
Poorest	105 (14.0)	119 (55.2)
Poorer	67 (8.7)	171 (54.3)
Middle	55 (6.6)	202 (55.8)
Richer	28 (2.9)	217 (48.4)
Richest	21 (1.9)	171 (34.5)
Occupation		
Not working	12 (1.9)	–
Professional	10 (1.9)	35 (33.6)
Clerical	0 (0.0)	14 (27.0)
Sales	16 (4.0)	52 (34.2)
Agriculture	169 (12.2)	343 (51.6)
Services	5 (5.0)	93 (40.3)
Skilled	36 (4.9)	214 (52.8)
Unskilled	28 (5.1)	129 (56.4)
Marital status		
Currently not married	104 (4.4)	420 (46.0)
Currently married	144 (7.7)	398 (48.2)
Formerly married	28 (19.8)	62 (64.3)

Computed from 2014 GDHS and 2014 LDHS

The results revealed that 10.2% of men aged 35–59, 21.7% of those with no education, 6.3% of men in rural areas and 27.8% of men who belong to the traditional/spiritual religious groups or who had no religion had used at least one type of tobacco in Ghana. By wealth status, occupation and marital status, 14% of respondents with poorest wealth status, 12.2% of agriculturalists and 19.8% of formerly married men had used at least one type of tobacco in Ghana. From Lesotho, 50.8% of men aged 35–39 had used at least one type of tobacco, 65.3% of those with no education, 51.3% of those in rural areas and 58.9% of those who belong to the traditional/spiritual religious groups or who had no religion had used at least one type of tobacco. Further results also showed that 55.8% of middle income men, 56.4% of unskilled men and 64.3% formerly married had used at least one type of tobacco in Lesotho (Table 1).

The prevalence of tobacco use was smaller in age group 15–24 years compared to the age groups 25–34 years and 35–59 years in both Ghana and Lesotho, although the association is stronger in Ghana. The AOR’s in Ghana are respectively 5.3 (95% CI: 3.29–8.59) and 9.7 (95% CI: 6.20–15.06), compared to respectively 1.7 (95% CI: 1.32–2.11) and 1.7 (95% CI: 1.36–2.12). The interaction between age and tobacco use in both countries showed a statistically significant relationship (*X* ^2^ = 1443.34 (5); *p* < 0.000). Smoking prevalence was smaller in men with higher level of education compared to men with no education in both Ghana and Lesotho, although the association was weaker in Ghana. The AOR in Ghana is 0.1 (95% CI: (0.02–0.11), compared to 0.2 (95% Cl: (0.17–0.30). The interaction between level of education and tobacco use in both countries indicated a statistically significant relationship (*X* ^2^ = 1595.04 (7); *p* < 0.000) (Table 2 and Additional file 1).

**Table 2:** Factors associated with tobacco use among males 15 years and older in Ghana (GDHS 2014) and Lesotho (LDHS 2014)

Variables	Ghana AOR (CI)	Lesotho AOR (CI)	*X* ^2^, (df) *P* value
Age			*X* ^2^ = 1443.34 (5); *p* < 0.000
15–24	1	1	
25–34	5.31(3.29–8.59)	1.67(1.32–2.11)	
35–59	9.67(6.20–15.06)	1.70(1.36–2.12)	
Level of education			*X* ^2^ = 1595.04 (7); *p* < 0.000
No education	1	1	
Primary	0.49(0.35–0.67)	0.69 (0.50–0.96)	
Secondary	0.19(0.14–0.25)	0.41(0.29–0.58)	
Higher	0.05(0.02–0.11)	0.19(0.17–0.30)	
Residence			*X* ^2^ = 1299.57 (3); *p* < 0.000
Urban	1	1	
Rural	2.13(1.67–2.73)	1.60 (1.31–1.95)	
Religion			*X* ^2^ = 1418.60 (7); *p* < 0.000
Christian	1	1	
Muslim	2.72(2.04–3.61)	1.05 (0.21–5.28)	
Traditional/spiritual/no religion	6.23(4.42–4.09)	1.73(1.21–2.47)	
Other	4.34(2.96–6.36)	1.07 (0.44–2.59)	
Wealth status			*X* ^2^ = 1424.01 (9); *p* < 0.000
Poorest	1	1	
Poorer	0.50 (0.37–0.68)	0.88 (0.64–1.22)	
Middle	0.39(0.28–0.55)	0.86(0.62–1.17)	
Richer	0.19(0.13–0.29)	0.70 (0.51–0.95)	
Richest	0.10(0.06–0.17)	0.39(0.51–1.12)	
Occupation			*X* ^2^ = 1422.30 (13); *p* < 0.000
Not working	3.93 (1.47–10.51)	–	
Professional	1	1	
Clerical	–	1.90 (0.90–3.61)	
Sales	2.68(1.08–6.53)	1.79 (1.05–3.07)	
Agriculture	9.28(4.54–18.99)	3.5(2.27–5.52)	
Services	5.21(1.89–14.34)	1.93(1.18–3.17)	
Skilled	3.45(1.58–7.54)	3.20(2.03–5.04)	
Unskilled	4.48(2.03–9.88)	3.89(2.36–6.39)	
Marital status			*X* ^2^ = 1328.04 (5); *p* < 0.000
Currently not married	1	1	
Currently married	0.77(0.57–1.04)	0.82(0.65–1.03)	
Formerly married	1.62 (0.99–2.28)	1.42 (0.89–2.28)	

Computed from 2014 GDHS and 2014 LDHS

*AOR* Adjusted Odds Ratio, *CI* Confidence Interval Reference category = 1, *df* degree of freedom

With place of residence, the prevalence of tobacco use was smaller among men in urban areas compared to rural areas in both Ghana and Lesotho, although the association is stronger in Ghana. The AOR in Ghana is 2.1 (95% CI: 1.67–2.73), compared to 1.6 (95% CI: (1.31–1.95). The interaction between place of residence and tobacco use in both countries showed a statistically significant relationship (*X* ^2^ = 1299.57 (3); *p* < 0.000). In both countries, prevalence of tobacco use was higher in men who are traditionalist/spiritualists or who had no religion compared to Christians, although the association was stronger in Ghana. In both countries, the interaction between religion and tobacco use showed a statistically significant relationship (*X* ^2^ = 1418.60 (7); *p* < 0.000). The AOR in Ghana is 6.2 (95% CI: (4.42–4.09) compared to 1.7 (95% CI: (1.21–2.47) in Lesotho. The prevalence of tobacco use was low among men with richest wealth status compared to men with poorest wealth status in both Ghana and Lesotho, although the association is weaker in Ghana. The AOR in Ghana is 0.1 (95% Cl: (0.06–0.17) compared to 0.4 (95% CI: (0.51–1.12). The interaction between wealth status and tobacco use in both countries indicated a statistically significant relationship (*X* ^2^ = 1424.01 (9); *p* < 0.000).

In relation to occupation, prevalence of tobacco use was smaller among professional workers compared to men in the Agricultural sector in both Ghana and Lesotho, although the association is stronger in Ghana. The AOR in Ghana is 9.3 (95% Cl: (4.54–18.99), compared to 3.5 (95% CI: (2.27–5.52). In both countries, the interaction between occupation and tobacco use showed a statistically significant relationship (*X* ^2^ = 1422.30 (13); *p* < 0.000). Formerly married men in both countries were more likely to use tobacco compared to currently not married men, although the prevalence was higher in Ghana. The AOR in Ghana is 1.6 (95% CI: (0.99–2.28)], compared to 1.4 (95% CI: (0.89–2.28) in Lesotho. The interaction between marital status and tobacco use in both countries indicated a statistically significant relationship (*X* ^2^ = 1328.04 (5); *p* < 0.000) (Table 2).

## Discussion

The study identified that there was a significant relationship between age and tobacco use in both countries. The odds of tobacco use was found to increase among the older age groups. Specifically, individuals who were in the older age group were more likely to use tobacco products as compared to those in the younger age group (15–24 years). This is consistent with a study from Nepal [34], Ghana [31], Brasil [35] and Madagascar [36]. This may be explained by the fact that older individuals have had a longer time to experience tobacco use and develop habits towards its use [37]. People who initiated smoking early in life have also been found to be less likely to quit smoking later in life [38]. Another possible explanation could be due to a lack of appropriate interventions for adults, which recalls the need for public health interventions that target this segment of population [30].

Our study also found that there was a significant relationship between level of education and tobacco use in both countries and the odds of tobacco use was found to decrease with level of education. Specifically, men with primary, secondary and higher level education were less likely to use tobacco compared to those with no education. This relationship between education and tobacco use is also consistent with previous studies in both developed and developing countries [21]. The explanation for this relationship is that the mechanisms through which education affect health behaviours is obvious. Education equips the individual with knowledge and skills to make informed and better health behaviour choices which positively affect the person’s health in the long run [31]. Therefore, men with higher level of education are more likely to make positive decision with regards to tobacco use. Place of residence also showed a significant relationship with tobacco use in both countries. Place of residence has also been found to predict tobacco use [21, 39, 40], although some of the studies have not found consistency in this prediction [41]. The findings that those in rural areas were less likely to use tobacco compared to urban dwellers except in Lesotho, shows the relationship between place of residence, education and occupation [31]. In addition, disparities in access to health information could account for the differences in tobacco use by place of residence [31].

There was a statistically significant difference in tobacco use across different religious groups in both countries. A number of studies have found differences in tobacco use by religious affiliations [30, 41]. Religious affiliation constitutes social network where not only social support exists but also behaviour is shared. Consequently, belonging to a religious sect that promotes health enhancing behaviours such as no smoking and non-excessive alcohol use motivates pursuing such lifestyles [31]. Men who were Muslims were more likely to use tobacco than Christians. This finding mirrors a number of studies conducted in Ethiopia [42] and in other countries [31, 34, 41]. Wealth status also showed a statistically significant relationship with smoking prevalence in both countries. Wealth, a measure of affluence in the study population, also showed the most outstanding gradient in tobacco use. The richer a man was, the less likely the tobacco use. The debate on why people with low income status are more likely to use tobacco has been resolved in several studies. One school of thought is that tobacco use is adopted by those in the lower socioeconomic groups as a way of coping with the stress, shame and humiliation that come with such status [43]. The above assertion could be explained in part why in poor countries like Ghana the poor are more likely to spend their income on tobacco compared to the rich [31].

Our study also found that there was significant relationship between occupation and tobacco use in both countries.. In both countries men in other occupational categories were more likely to use tobacco compared to professional workers. The possible justification could be the ethics associated with professional work that might prevent professional workers from tobacco use. Schools and some offices may have internal law which ban tobacco use [30]. A study in Nepal found that adults in manual occupations were more likely to use tobacco as compared to professional/clerical service jobs [34]. Type of occupation was also significantly associated with tobacco use in Madagascar [36].

This study also found that there was a significant relationship between marital status and tobacco use in both countries. Specifically, currently married men were less likely to use tobacco compared to currently not married men. This could be explained by the fact marriage takes away some of the stressful situations that may lead to tobacco use. Again, marriage is seen as a means through which partners regulate the health behaviours of each other. Obviously, it is expected that men who are currently married will be less likely to use tobacco However, formerly married men were more likely to use tobacco compared to currently not married men. This finding could be attributed to the relationship between divorce and health behaviour. It is expected that formerly married men in their bid to overcome the consequences of divorce or death of a partner would resort to tobacco use.

## Conclusion

Although similar socio-economic inequality factors provided an understanding of tobacco use among men in Ghana, and Lesotho, there were variations in relation to how each socio-economic inequality variable influences tobacco use. However, in both countries, there was a significant relationship between all the socio-economic variables and tobacco use. The odds of tobacco use were high among men in the older age groups, low educational level and Muslims and traditionalists. There was also high prevalence of tobacco use among men whose occupation were clerical sales, agriculture, services, skilled and unskilled as well as formerly married men. Therefore, these factors need to be considered for specific public health interventions to reduce tobacco use in Ghana, and Lesotho. However, tobacco use was low in rural areas and decreased among those with higher wealth status, those with no occupation and currently married men.

## Additional file

Additional file 1: Table S1. Multivariate analysis of the factors associated with tobacco use among males 15 years and older in Ghana (GDHS 2014) and Lesotho (LDHS 2014). (DOCX 13 kb)
