# Supplementary material for: Understanding tobacco use and socioeconomic inequalities among men in Ghana, and Lesotho
Source: Arch Public Health. 2017 Jul 3;75:30. doi: 10.1186/s13690-017-0197-5 (PMC5494801; doi:10.1186/s13690-017-0197-5)
Supplement: Additional file 1: Table S1. — Multivariate analysis of the factors associated with tobacco use among males 15 years and older in Ghana (GDHS 2014) and Lesotho (LDHS 2014). (DOCX 13 kb) [file 13690_2017_197_MOESM1_ESM.docx]

Table S1: Multivariate analysis of the factors associated with tobacco use among males 15 years and older in Ghana (GDHS 2014) and Lesotho (LDHS 2014)

| Variables | Ghana  OR (CI) | Lesotho  OR (CI) |
| --- | --- | --- |
| Age |  |  |
| 15 – 24 | 1 | 1 |
| 25 – 34 | 7.63(4.40 – 13.25) | 1.94(1.47 – 2.58) |
| 35 – 59 | 13.30(7.58 – 23.32) | 1.84(1.38 – 2.48) |
| Level of education |  |  |
| No education | 1 | 1 |
| Primary | 0.55(0.38 – 0.78) | 0.74 (0.53 – 1.04) |
| Secondary | 0.32(0.22 – 0.45) | 0.52(0.35 – 0.76) |
| Higher | 0.19(0.07 – 0.50) | 0.32(0.18 – 0.55) |
| Residence |  |  |
| Urban | 1 | 1 |
| Rural | 0.79 (0.56 – 1.12) | 1.02 (0.79 – 1.32) |
| Religion |  |  |
| Christian | 1 | 1 |
| Muslim | 1.66(1.20 – 2.31) | 1.93 (0.36 – 10.36) |
| Traditional/spiritual/ no religion | 2.75(1.85 – 4.09) | 1.41 (0.98 – 2.04) |
| Other | 2.56(1.72 – 3.83) | 0.92 (0.37 – 2.31) |
| Wealth status |  |  |
| Poorest | 1 | 1 |
| Poorer | 0.78 (0.55 – 1.09) | 1.01 (0.73 – 1.41) |
| Middle | 0.67(0.43 – 1.02) | 1.07 (0.76 – 1.49) |
| Richer | 0.40(0.22 – 0.70) | 1.01 (0.71 – 1.44) |
| Richest | 0.32(0.16 – 0.65) | 0.76 (0.51 – 1.12) |
| Occupation |  |  |
| Not working | 1.84 (0.65 – 5.29) | - |
| Professional | 1 | 1 |
| Clerical | - | 1.26 (0.61 – 2.58) |
| Sales | 1.61 (0.60 – 4.35) | 1.14 (0.65 – 2.01) |
| Agriculture | 2.23(0.95 – 5.22) | 1.72(1.04 – 2.83) |
| Services | 2.71(0.89 – 8.24) | 1.31 (0.77 – 2.22) |
| Skilled | 1.82 (0.76 – 4.38) | 1.90(1.16 – 3.11) |
| Unskilled | 2.26(0.93 – 5.54) | 2.18(1.27 – 3.73) |
| Marital status |  |  |
| Currently not married | 1 | 1 |
| Currently married | 0.41(0.29 – 0.57) | 0.86 (0.67 – 1.09) |
| Formerly married | 1.21 (0.71 – 2.07) | 1.30 (0.80 – 2.12) |
|  |  |  |

Computed from 2014 GDHS, 2014 KDHS, and 2014 LDHS

OR= Odds Ratio CI= Confidence Interval Reference category = 1
